# Editorial: Methods for imaging and omics data science: advances, applications, and spatiotemporal innovations

**DOI:** 10.3389/fgene.2026.1922890

**Published:** 2026-07-29

**Authors:** Himel Mallick, Lingling An, Shrabanti Chowdhury, Anchal Ghai, Suvo Chatterjee, Siyuan Ma, Ali Rahnavard, Pinaki Sarder

**Affiliations:** 1 Division of Biostatistics, Department of Population Health Sciences, Weill Cornell Medicine, Cornell University, New York, NY, United States; 2 Division of Gastroenterology and Hepatology, Weill Cornell Medicine, New York, NY, United States; 3 Department of Statistics and Data Science, Cornell University, Ithaca, NY, United States; 4 Interdisciplinary Program in Statistics and Data Science, The University of Arizona, Tucson, AZ, United States; 5 Department of Epidemiology and Biostatistics, The University of Arizona, Tucson, AZ, United States; 6 Department of Biosystems Engineering, The University of Arizona, Tucson, AZ, United States; 7 Department of Genetics and Genomic Sciences, Icahn School of Medicine at Mount Sinai, New York, NY, United States; 8 Department of Biomedical Engineering, University of Texas Southwestern Medical Center, Dallas, TX, United States; 9 Department of Epidemiology and Biostatistics, Indiana University School of Public Health, Bloomington, IN, United States; 10 Department of Biostatistics, Vanderbilt University Medical Center, Nashville, TN, United States; 11 Department of Biostatistics and Bioinformatics, Computational Biology Institute, Milken Institute School of Public Health, The George Washington University, Washington, DC, United States; 12 Division of Medicine – Quantitative Health, University of Florida, Gainesville, FL, United States

**Keywords:** artificial intelligence - AI, biostatistics, computational biology and bioinformatics, digital pathology, imageomics, machine learning, multimodal AI, omics data science

Computational biology is being reshaped by foundation models and multimodal artificial intelligence (Khan et al., 2025; Acosta et al., 2022). Large models pretrained across sequences, single-cell profiles, tissue images, and spatial context are beginning to learn shared representations that cut across data types, and they are rapidly changing how omics and imaging data are analyzed and combined (Zhou et al., 2025; Mollerus et al., 2026; Baghbanzadeh et al., 2026). This shift makes the methodological questions at the heart of this Research Topic more pressing (Kasireddy et al., 2026; DenAdel et al., 2026; Wang, 2025). Foundation models are only as trustworthy as the statistical principles, benchmarks, and domain-aware modeling choices that underlie them, and the spatial, temporal, and multimodal structure of modern biomedical data continues to demand purpose-built methods for representation, inference, and integration. By assembling rigorous statistical and computational advances at the interface of imaging and omics, this Research Topic offers a timely foundation for the multimodal, AI-driven analyses that are now routine in biomedicine.

This Research Topic is the third and concluding installment of a trilogy curated for *Frontiers in Genetics*. The first volume focused on statistical and computational methods for microbiome multi-omics data (Mallick et al., 2020), the second broadened the lens to methods spanning single-cell and microbiome sequencing data (Mallick et al., 2022), and the present volume extends the scope further still, to the wider landscape of imaging and omics data science, with an emphasis on spatial and spatiotemporal innovation. The arc of the trilogy mirrors the trajectory of the field itself, moving from single-modality sequencing toward the integrated, spatially aware, and multimodal analyses that increasingly define modern biomedical research.

Imaging and omics technologies developed largely in parallel, yet they have converged on remarkably similar analytical challenges (Jennings et al., 2026; Vavekanand, 2026). Both produce data that are high-dimensional, noisy, heterogeneous, sparse, and shaped by platform-specific technical variability, and both increasingly require explicit modeling of spatial, temporal, and spatiotemporal dependence within and across modalities. These shared properties have allowed methods to migrate productively between domains, with techniques developed for sequencing data adapted (with appropriate modifications) for imaging data and the reverse. This crosstalk is precisely the exchange the trilogy was designed to encourage.

This Research Topic comprises eleven articles (including this editorial): three Methods papers, six Original Research papers, and one Review. The contributions span the full analytical pipeline, from upstream representation and feature construction to downstream modeling, inference, and integration. To highlight the coupling between fields, we group the papers into four complementary themes: (i) spatially resolved transcriptomics, (ii) imaging, spatial pathology, and radiomics, (iii) single-cell and multi-omics integration, and (iv) sequence-level genomics, phylogenetics, and regulatory biology. Many of these methods are domain-specific by design, yet a recurring feature of the Research Topic is the potential for techniques to transfer across the imaging and omics divide.

A central theme of this volume is the analysis of spatially resolved transcriptomics (SRT), where preserving tissue context is essential for biological interpretation. Spatial domain identification, the task of partitioning tissue into coherent regions, is a foundational step that remains sensitive to noise and to the choice of spatial neighborhood. Peng et al. address this with DWGCN, a distance-weighted graph convolutional network that incorporates spatial proximity directly into the graph construction to deliver more robust spatial domain identification across SRT platforms. Complementing the domain-level view, Yang et al. develop a Bayesian hidden Markov interaction model for detecting spatially variable genes in imaging-based SRT, accommodating the point-referenced, single-molecule structure characteristic of these assays. Moving from individual genes to intercellular signaling, Ma et al. introduce RECCIPE, a framework for assessing how localized cell-cell interactions shape gene expression in multicellular spatial transcriptomics data, providing a principled route to dissect the spatial determinants of expression heterogeneity.

The imaging side of the Research Topic is anchored by methods for spatial pathology and radiomics, where the analytical unit shifts from sequenced features to image-derived measurements. Osher et al. propose SPARTIN, a Bayesian method for quantifying and characterizing cell-type interactions in spatial pathology data, formalizing the spatial co-organization of cells within tissue. In the radiology setting, Shoemaker et al. develop a Bayesian feature selection approach for radiomics that incorporates feature reliability metrics, an important consideration given the well-documented sensitivity of radiomic features to acquisition and segmentation variability. Together these contributions illustrate how the statistical machinery familiar from omics analysis, including hierarchical Bayesian modeling and principled variable selection, transfers naturally to imaging-derived data.

Integration across modalities, both within omics and between omics and imaging, forms a third theme. Ellis et al. present jrSiCKLSNMF, a joint graph-regularized non-negative matrix factorization method for clustering single-cell multimodal omics data, leveraging shared structure across modalities to recover biologically coherent cell populations. At the bulk level, Wang J. et al. apply multi-kernel learning to integrate multi-omics measurements for hepatocellular carcinoma subtyping, demonstrating how kernel-based integration can combine heterogeneous data types into clinically meaningful patient strata. These papers underscore that integrative modeling, whether at single-cell or sample resolution, remains a key driver of biological insight.

Finally, several contributions extend the Research Topic toward sequence-level genomics, phylogenetics, and regulatory biology. Wang T. et al. introduce CGRWDL, an alignment-free phylogeny reconstruction method for viruses that combines chaos game representation with a dynamical language model, offering a scalable alternative to alignment-based approaches for rapidly evolving genomes. Sousa et al. report a detailed identification, taxonomy, and comparative genomics analysis of nine *Bacillus velezensis* strains, connecting genomic features to traits of biotechnological relevance. Rounding out the volume, de Faria Poloni et al. provide a Review of long non-coding RNAs, arguing that subcellular localization is a key determinant of their regulatory function and synthesizing the current understanding of how spatial context governs lncRNA activity.

Several of the methods in this volume are accompanied by open-source software, providing a durable resource for the community and reinforcing the field’s movement toward reproducible and interpretable analysis. As this trilogy concludes, we expect the boundary between imaging and omics to continue dissolving: through tighter coupling of molecular and morphological measurements, richer spatial and spatiotemporal models that link tissue architecture to molecular state, and foundation models that learn jointly across modalities while remaining grounded in sound statistical reasoning. We hope this Research Topic, and the trilogy it completes, offers a shared methodological vocabulary that helps catalyze that convergence. We are grateful to all the authors for their contributions, to the reviewers for their careful evaluations, and to the Frontiers editorial staff for their support in assembling this Research Topic.
